# Rheumatic Fever

**Published:** 1901-01-12

**Authors:** 


### Hospital Clinics and Medical Progress.

### RHEUMATIC FEVER.

From time to time the most diverse views have been held in regard to the nature of acute rheumatism or rheumatic fever, but all are agreed as to its gravity and importance. For many years the tendency was all in the direction of seeking for the cause of this disease in some abnormal constituent in the blood; of late the drift has all been towards the microbe, and towards including rheumatism among the acute infections; and so there has seemed to be, as indeed there has been, a wide divergence of opinion. But with the general recognition of the fact that the symptoms of admittedly infectious diseases are largely due to toxins produced by the specific micro-organisms associated with them, the path has been made easy for both schools to come into the same fold, and, while admitting that there is a condition of blood by which many of the symptoms can be produced, to look for a microbic infection as the origin of the whole disease.

### Dampness and Rheumatic Fever.

Having got so far in cutting ourselves adrift from old beliefs we are now asked to put aside the time-honoured tradition that dampness is to be counted among the causes of rheumatic fever. Dr. Arthur Newsholme,[1] writing on this subject, says that the confusion between the chronic forms of "rheumatism" and rheumatic fever is probably the chief cause of this old and serious error. It has long been recognised that among other signs of on-coming rain is the pathological one that

"Old Betty's joints are on the rack,"

and on the idea that the joint pains which are made worse by bad weather are "rheumatic" in character, it is assumed that acute rheumatism must also be worse in damp weather, and as a further assumption must be more common among those living on damp soil. But all this is wrong. "The primary error consists in assuming that there is any pathological or etiological relationship between the heterogeneous diseases known as chronic rheumatism and the specific febrile disease, rheumatic fever." From his investigations he is able to show that when epidemics of acute rheumatism occur they take place in years in which the soil is exceptionally dry, and the ground water exceptionally low.

### The Pathology of Rheumatic Fever.

In his view it seems most feasible to regard rheumatic fever as not directly infectious from case to case, but as an essentially soil disease, due to a saprophytic soil organism which is "drowned ont" in wet years, multiplies rapidly in dry years, and is transferred to the human recipient by means as yet unknown, possibly by domestic vermin, or maybe by flies conveying it to milk and other kinds of food. If, then, we accept the dipplococcus described by Dr. F. J. Poynton[1] as the microbic cause of the disease, we may picture rheumatic fever as an infective disease, occurring among people predisposed to it, the microbe by which it is caused usually gaining access by way of the tonsil, circulating in the blood, and occasioning local lesions, such as nodules, inflammations of connective tissues, arthritis, endocarditis, pericarditis, &c., in different parts of the body. We may further suppose that in the local lesions the cocci are destroyed, but in many cases not without damage having been done to the important tissues in which they have become localised. Thus we see a disease which although acute and severe runs a favourable course so far as life is concerned, but nevertheless leaves permanent traces of its occurrence in the form of chronic valvular disease and pericardial adhesions. Reviewing the relation between rheumatic fever and the throat Dr. St. Clair Thomson[1] says that a proportion varying from 30 to 80 per cent. of the cases of acute rheumatism are preceded by an angina, and that the tonsil may be the port of entry of the rheumatic virus, even although the naked-eye appearance of the throat may give no indication of its being affected.

### Rheumatism and the Heart.

By far the most serious effects of rheumatism are those which fall upon the heart. Writing upon this subject Dr. G. A. Gibson[1] points out that the rheumatic poison may affect not only the pericardium and the endocardium, but the muscle itself, and he agrees with Poynton in considering all these inflammations as of microbic origin. Starting then with the conception that the cardiac lesions are part of the rheumatic infection, and knowing what results may be expected should the infective material localise itself upon the heart, we are prepared to see how important it is, with a view to obviate implication of that organ, to enjoin rest in the highest degree available which of necessity involves rest of the body. The heart must work, but it is well to remember that by ensuring an absence of muscular exertion the force and the frequency of the action of the heart, the friction between the inflamed pericardial surfaces, the shock of the closure of the valvular cusps, and the strain upon the muscular fibres may be enormously reduced. From the first moment, therefore, of an attack of acute rheumatism until some days after every symptom has finally disappeared the patient should be compelled to retain a horizontal position. It is important also that the diet should be of an unstimulating character. During the earlier periods of acute rheumatism the best form of diet is one composed mostly of milk, but as time goes on soups, farinaceous foods, and the lighter forms of flesh may be introduced.

### Acute Dilatation of the Heart.

Dr. D. B. Lees[2] points out the importance of ascertaining the size of the heart by careful percussion and palpation in cases of diphtheria, influenza, and rheumatic fever. In all these diseases, but especially in diphtheria, sudden death is apt to occur as a result of inflammation and degeneration of the cardiac muscle, and in all of them dilatation of the heart is to be taken as the sign indicating the existence of this degeneration. This dilatation is not nearly so dangerous a symptom in rheumatism as in diphtheria, but its dependence upon an inflammatory change in the muscle substance indicates a connection between acute rheumatism and other more obviously microbic infections which cannot be overlooked. "It cannot be questioned," says Dr. Lees, "that in diphtheria and in influenza the injury to the heart is caused by the deleterious action of the toxin produced by a microbe. In each of these two diseases the microbe is well known. Is it not then in the highest degree probable that rheumatic fever also is a microbic disease, and that the resulting cardiac inflammation is due to its toxin?"

Among the symptoms of cardiac dilatation on which Dr.

Lees specially insists are those which can be ascertained by percussion and palpation. The stethoscope, he says, has been far too dominant in the physical examination of the heart, and palpation and percussion are often most inefficiently performed. This is especially the case with percussion, which when it is confined to the ascertainment of what is called the "superficial cardiac dulness" gives no information as to the size of the heart. To find out the size of the heart the percussion should be light and the terminal phalanx should by preference be used as a pleximeter so that the finger may be kept away from the chest wall excepting over the part which is being percussed. With a little practice there should be no difficulty in defining with considerable accuracy the limit of the heart both to left and to right. Such careful diagnosis of the condition of the heart cavities is of great importance to the patient. Dilatation and feebleness of the left ventricle may indicate urgent danger of death from syncope, and neglect of this indication may cost the patient's life. Dilatation of the right auricle (quite easily detected by percussion in the fourth right interspace) and weakness of the right ventricle (detected by palpation of the epigastric region) are usually accompanied by considerable dyspnœa and often by some lividity, while a marked degree of dilatation of the right auricle may indicate grave danger of death from asphyxia, and call urgently for venesection or leeches. Dr. Lees says that his experience as an examiner in medicine forces him to the conclusion that these facts are very inadequately recognised. Candidates feel for the cardiac impulse as if that were equivalent to the left border of the heart, while the right limit of the heart he usually neglects altogether, and "even some physicians of great eminence are apparently not conversant with the fact that the dulness of the right auricle normally extends one fingerbreadth into the fourth right space, and that its border can be easily detected by careful light percussion."

### Medicinal Treatment of Rheumatic Fever.

With regard to drugs there appears good ground for the belief that the salicyl series considerably diminishes the cardiac effects of rheumatism. Dr. Gibson[1] says that these drugs must therefore be continued in full doses until every symptom has disappeared—until the pulse and temperature have become normal, and every appearance of joint affection has subsided—and even after that it is well to go on with them for some time. Then absorbents may be begun, the most satisfactory one being iodide of sodium, 10 to 15 grains being given three times a day. This may be continued for some weeks, being combined, if there be any appearance of anæmia, with iodide of iron. During all this time no remedy that can stimulate the heart, such as digitalis or strophanthus, should be given, the whole aim being to maintain the heart's action at a low level. Afterwards, however, cardiac tonics may be commenced. It is well to remember to give a mercurial aperient every few days. It would be entering into the land of speculation to attempt to explain how blisters act, but that they do good there seems to be no doubt. Dr. Gibson recommends the application of small fly-blisters every night or every second night over the præcordia and their neighbourhood. Dr. Luff[1] says that the danger in connection with the use of salicylates is that they so rapidly relieve the urgent symptoms—the articular pain and the fever—that there is a tendency to discontinue their use at too early a stage and too suddenly, and that the patient is therefore not kept at rest and on liquid diet for as long a period as he would be under other forms of treatment. That is the answer to the statement that relapses have become more frequent since the employment of salicylates, if the statement is true, which according to Dr. Luff's experience is not the case. Alkalies, however, are also useful, and Dr. Luff says that the disease is most successfully treated by giving a combination of a salicyl compound with an alkaline bicarbonate, such as 20 grains of sodium salicylate and 30 grains of potassium bicarbonate every two hours until the pain is relieved, when the same quantities may be continued every four hours until the temperature has fallen to normal, when a still further reduction may be made, and carried on until all joint symptoms have disappeared. The occurrence of deafness, noises in the ear, and possibly delirium are indications that the salicylate has been pushed too far, and that it should be diminished or even withdrawn. The probability of these effects being produced is, however, much lessened by producing a free action of the bowels at the outset by a dose of calomel, followed by a saline purge. Dr. Luff specially insists upon prolonged treatment before patients are allowed to dispense with rest and salicylates. It must be borne in mind, he says, that in cases of rheumatic endocarditis the vegetations may disappear, and that complete resolution of the inflamed valves is possible. This we think is a very important point, and one far too seldom insisted on. To effect this resolution full doses of salicylates and alkalies must be continued for some time after all arthritis has disappeared, and absolute rest must be enforced for six or eight weeks after the disappearance of the joint symptoms.

For the relief of pain Dr. Luff recommends, among other things, the use of salicylate of methyl, applied on lint over the affected joint, the whole being covered with gutta-percha tissue, the edges of which are sealed down by wetting with a little chloroform. Writing on the same subject, Dr. Alfred Stengel,[3] of Philadelphia, also advises the local employment of this preparation; but he applies it in the form of a 10 per cent. or a 20 per cent. ointment covered with lint and gutta-percha. In some very acute inflammatory cases, after applying this, he fixes the whole joint in plaster of Paris.

### Rheumatism in Childhood.

Dr. Still[1] says that the symptoms of rheumatism in childhood differ from those in adults chiefly in the much slighter character of the joint manifestations and in the much greater prominence and frequency of the heart affections; and this is to be borne in mind, that with these little patients pains in the limbs may be just as significant of rheumatism as swollen, red, and tender joints, and just as liable to be accompanied by cardiac affections. "The practical importance of recognising the significance of these slight pains in the limbs of children—'growing pains' as they are too often called—can hardly be overrated. They may be the earliest indication of rheumatic taint, and as such should always serve as a danger signal," for it seems probable that active treatment in this early stage may not only cause the disappearance of the pains, but may also prevent those affections of the heart which are so distressing a feature in the rheumatism of childhood. The presence of these indefinite pains must always lead us to keep a careful watch upon the heart. Stiff neck, pain in the hip, and pain at the back of the knee are mentioned as examples of what may be the first indications of acute rheumatism in children, while the importance of the rheumatic nodule as often presaging severe endocarditis is insisted on. The occurrence of such nodules is almost conclusive proof of rheumatism.

### Red Hair and Rheumatism.

Among the various warning signs given by Dr. Still as leading one to look out for rheumatism we will mention two. One is that the rheumatic child is *par excellence* the nervous child. Excitability, timidity, shyness, night terrors, somnabulism, habit spasms, such as blinkings and twitchings, are indications of this nervous disposition. The other warning sign is red hair. He suggests that this colour of the hair may be only the index of some fine peculiarity, perhaps in the chemistry of metabolism, which produces a soil favourable to rheumatic infection; at any rate it is one of the many little indications which are sometimes of value in leading to the early detection of rheumatism in a child. Dr. Still says, "In four days there were among my out-patients eleven children with red hair. Of these two were attending with articular rheumatism; one had occasional pain and swelling in the knees: his mother had red hair and frequent pains in the limbs, and her brother and sister had 'rheumatic fever'; one had 'pains in the knees' and his mother had 'rheumatism'; three others showed a history of 'rheumatic fever' in the mother or father; one was attending with chorea; one had a brother attending with articular rheumatism; only two out of the eleven showed no rheumatism or chorea in themselves or their families." Certainly this seems more than a coincidence, and every little thing that helps us in the early detection of rheumatism in the child is of importance. No condition, says Dr. Still, can be more distressing than that of the child dying of cardiac rheumatism, and we would add that nothing can well be more exasperating than the discovery, just when the adolescent is budding into productive manhood, that he must be hampered and kept back in everything that he attempts by a cardiac defect which has remained to him as the memento of some undiscovered and untreated rheumatism in childhood.

[1] Practitioner, January 1901. [2] Brit. Med. Jour., Jan. 5. [3] Medical News, Dec. 22.

